# Enhanced Metal Surface Processing Through the No-Stray-Corrosion Controllable Electrolyte DistributionElectrochemical Machining Method Utilizing a Water-Absorbent Porous Ball

**DOI:** 10.3390/mi16070822

**Published:** 2025-07-18

**Authors:** Jiankang Wang, Qiyuan Cao, Ye Chen, Wataru Natsu, Jianshu Cao

**Affiliations:** 1 School of Engineers, Beijing Institute of Petrochemical Technology, Qingyuan North Road No. 19, Daxing District, Beijing 102617, China; wangjiankang@bipt.edu.cn; 2 Academy of Artificial Intelligence, Beijing Institute of Petrochemical Technology, Qingyuan North Road No. 19, Daxing District, Beijing 102617, China; 2023312187@bipt.edu.cn; 3 General Technology Group Machine Tool Engineering Research Institute, Wangjing Road No. 4, Chaoyang District, Beijing 100102, China; chenye11@gt.cn; 4 Department of Mechanical Systems Engineering, Tokyo University of Agriculture and Technology, 2-24-16 Nakacho, Koganei City, Tokyo 184-8588, Japan; summer@cc.tuat.ac.jp

**Keywords:** metal surface processing, no-stray-corrosion ECM method, controllable electrolyte distribution ECM (CED-ECM), water-absorbent porous ball, modeling and simulation

## Abstract

The Electrochemical Machining (ECM) method is one of the most widely used processing methods in metal surface processing, due to its unique advantages. However, the electrolyte in ECM causes stray corrosion on the workpiece. To overcome these shortcomings, we have developed a no-stray-corrosion ECM method called the controllable electrolyte distribution ECM (CED-ECM) method. However, its practical application in metal surface processing remains largely unexplored. In this study, to improve the CED-ECM method, we delved deeper into the aforementioned aspects by simulating the actual ECM process using COMSOL Multiphysics and rigorously validating the simulation results through practical experimental observations. Then, our efforts led to the application of the CED-ECM method to metal surface processing for the SUS304 workpiece, producing noteworthy results that manifest in diverse cross-sectional profiles on the processed surfaces. This research demonstrates a validated simulation framework for the CED-ECM process and establishes a method for creating user-defined surface profiles by controlling pass intervals, enabling new applications in surface texturing.

## 1. Introduction

In industrial production, the surface characteristics of metal components often undergo changes due to the influence of processing methods [1]. For instance, cutting forces and heat can lead to the formation of micro-cracks, surface oxidation layers, and high-temperature tempering layers [2,3,4]. Additionally, alloying elements in metal materials may undergo phase transformations during heating or cooling, resulting in metamorphic layers on the metal surface [5,6]. These alterations in the surface characteristics of metal materials can have adverse effects on the subsequent processing and utilization of metal components.

Therefore, metal surface processing plays a crucial role in the manufacturing industry, serving as the fundamental stage for shaping and refining metal components to meet specific design requirements. This process encompasses various techniques, including but not limited to grinding, electrical discharge machining (EDM), and ECM, all aimed at achieving the desired shape, size, and surface characteristics of metal components.

Qiao et al. [7] reported the use of grinding in processing titanium alloy materials produced by laser melting. Their results demonstrate that by integrating research on material surface roughness, geometric shape, and microstructure, along with optimizing grinding process parameters, the surface quality of TiC-reinforced titanium matrix composite (TMC) materials can be significantly enhanced. In a similar vein, Rakurty et al. [8] employed lower grinding parameters and appropriate cutting fluids to improve the surface integrity of additive manufacturing-produced titanium alloys (Ti6Al4V) during the finishing process, resulting in improved surface quality and processing efficiency. This approach also placed emphasis on reducing residual stress after processing. Perná et al. [9] employed a grinding wheel containing geopolymer-based substances in metal grinding processing, enhancing processing accuracy and efficiency due to the material’s hardness and wear resistance.

However, the continuous wear of the grinding wheel during the grinding process negatively impacts the quality and efficiency of the overall process. Simultaneously, the grinding process generates a significant amount of heat, leading to potential defects such as stress deformation and surface cracks on the workpiece. Additionally, high-precision machine tools are often required for grinding processes, contributing to a notable increase in processing costs [10].

In contrast to grinding, the EDM method is a non-contact processing method. First, the EDM method utilizes high temperatures during the discharge process to melt/gasify the processed material. Guan et al. [11] replaced traditional diamond wheels with a rotating cup wheel as the tool electrode to achieve discharge grinding. This approach enabled high-precision grinding of a SiC wafer with a thickness of 30 μm and a diameter of 20 mm, achieving a subsurface damaged layer of <1 μm. Furthermore, in an effort to merge the advantages of grinding and EDM, Yan et al. [12] utilized sintered diamond as an electrode tool in EDM for grinding SiC. The results indicated that this method exhibited high processing efficiency, low cost, reduced surface roughness, and stable chemical composition. Furthermore, the laser surface processing method is another advanced method which is usually used in metal surface processing, especially in metal hydrophilic/hydrophobic surfaces [13,14] and surface micro-patterns [15].

However, no matter whether the EDM method or the laser processing method is used, they both have their limitations. For example, the high temperatures generated during processing result in a thermal influence zone at the workpiece processing site, causing recast layers, micro-cracks, and other effects that will impact the material’s fatigue strength [16].

In contrast, during metal surface processing, ECM stands out as a common method due to its advantages of no tool wear, no residual stress, no heat-affected layers, and no cracks or burrs [17]. When compared with other processing methods like grinding and EDM, these advantages make ECM highly suitable for metal surface processing.

Therefore, Adrian et al. [18] utilized the Jet-ECM method with a 50 mm × 0.3 mm slit nozzle in metal surface processing to achieve surface texture on stainless steel. The research results demonstrate that the Jet-ECM method can process a stainless steel workpiece with multi-scale surface characteristics, offering the ability to customize production based on specific application surface characteristics. It is widely known that a flowing electrolyte is necessary during the common ECM process. However, as reported by other researchers [19,20], electrolyte suction tends to distribute not only in the target processing area on the workpiece surface but also in the unneeded processing area, leading to stray corrosion.

Hence, Wu et al. [21,22,23] proposed a novel Mask Electrolyte Jet Machining (MEJM) method. By combining a duckbill-shaped electrolyte jet nozzle with Mask Electrochemical Machining (MECM) technology, this method retains the controllable flow field characteristics of traditional Jet-ECM while introducing a mask constraint mechanism: a pre-patterned mask with hollow structures is covered on the workpiece surface. During processing, the electrolyte is only sprayed through the hollow regions onto the workpiece surface, and electrochemical reactions are confined to these hollow areas. The mask-covered regions remain unaffected due to physical isolation from the electrolyte. This selective processing characteristic enables MEJM to efficiently fabricate microstructures with specific geometric shapes or matrix-arranged patterns, providing a new approach to the precise fabrication of complex micro-patterns.

In our prior research [24,25,26], to prevent stray corrosion and maintain precise control over electrolyte distribution, we employed a Maifan Stone ball (MFS-ball), a porous solid sphere used as the electrolyte absorption material in ECM. In this study, the MFS-ball, which was bought from markets, consists of clay, Maifan stone, and other materials [27]. Leveraging the capillary principle, the electrolyte is drawn into the minute pores on both the interior and exterior of the MFS-ball, forming a thin, uniform electrolyte film on the MFS-ball’s surface. Due to the influence of liquid surface tension, the electrolyte is confined to the immediate vicinity of the contact point between the workpiece and the MFS-ball, preventing its random spread over the workpiece surface. This unique approach sets the controllable electrolyte distribution ECM method apart from conventional ECM methods relying on the flowing electrolyte. After the ECM process, there is no trace of stray corrosion on the workpiece surface. Additionally, the impacts of the primary experimental parameters on the ECM processing results were thoroughly examined through numerous practical experiments.

After that, in order to gain insights into the machining dynamics within the ECM processing region, we employed finite element analysis software, COMSOL Multiphysics 6.1, to simulate and analysis the 2D model of the ECM process [26]. Specifically, during the simulation, we examined a cross-section perpendicular to the direction of MFS-ball movement within the ECM process, providing a comprehensive understanding of factors such as the voltage distribution, the current density distribution, and the processed depth. However, the 2D model fails to capture the full dynamics of the entire ECM process, limiting predictive accuracy for complex geometries.

In this paper, we firstly used COMSOL Multiphysics to conduct a detailed simulation of the entire ECM process, obtaining much more detailed and real voltage distribution, current distribution, and the overall ECM result on the workpiece surface. Subsequently, to validate the simulation results, we compared them with actual experimental results. It was found that the simulation results closely aligned with the experimental results, establishing excellent mutual credibility between them. Finally, in terms of the metal surface processing, by adjusting the interval between every two ECM processes, we obtained processed results with various cross-section profiles, such as the profile with a flat bottom and profiles with step-like structures, providing a clearer guide and direction for future practical applications and research.

## 2. Experimental Devices, Processing Mechanism, and Experimental Operation for Metal Surface Processing

### 2.1. Experimental Devices

**Figure 1:**
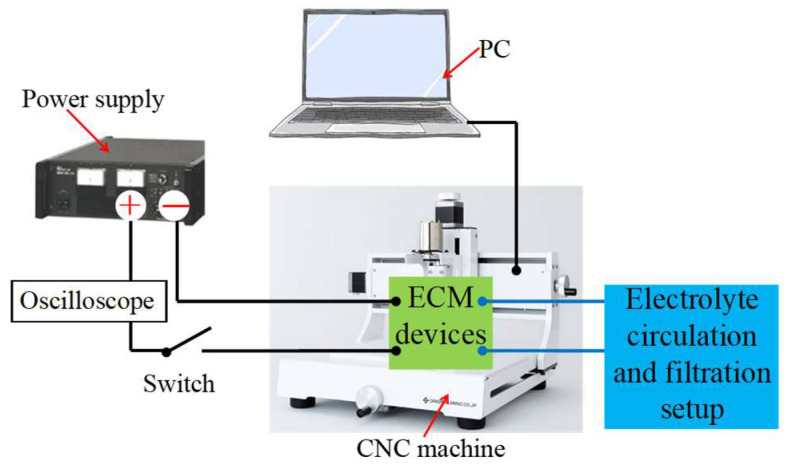
Experimental setup.

**Figure 2:**
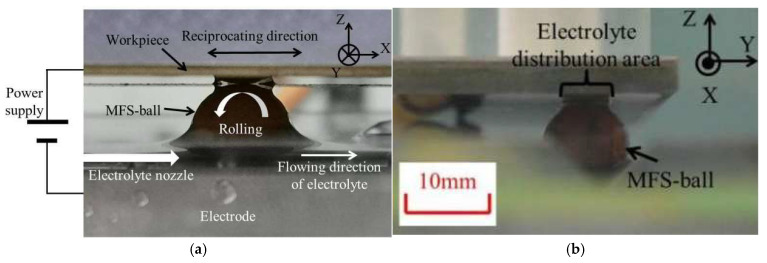
ECM setup and the ECM processing photo. (**a**) ECM processing photo view parallel to the scanning direction. (**b**) ECM processing photo view perpendicular to the scanning direction.

The complete experimental setup utilized in this research is depicted in Figure 1. The whole process was conducted using a CNC machine (KitMill CL420, ORIGINALMIND Inc., Tokyo, Japan) in conjunction with a PC, serving as the drive and control device. A DC power supply (BWS 120–2.5, TAKASAGO Ltd., Takasago, Japan) powered the ECM process. An oscilloscope (WF1973, NF Corp., Yokohama, Japan) was employed to measure the voltage and current waveforms during ECM, and two pumps (GPU-1, AS ONE Corp., Osaka, Japan) circulated the electrolyte in the electrolyte circulation and filtration setup. As shown in Figure 2, both the ECM setup and a photo of the processing during the experiment are presented. In the ECM section, a workpiece (SUS304), an electrode (SUS304), and an MFS-ball were positioned between the workpiece and the electrode.

**Figure 3:**
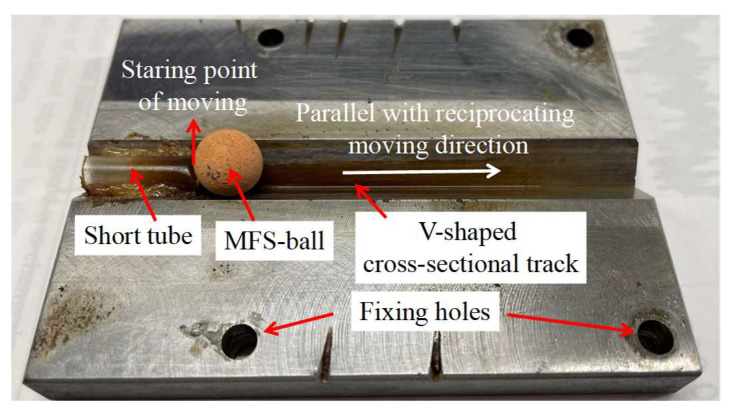
Tool electrode.

As illustrated in Figure 3, the tool electrode features a V-shaped cross-sectional track on its surface, mechanically constraining the MFS-ball’s motion along this track. The starting point of the MFS-ball’s movement is located at one end of the short tube, which also serves as the electrolyte entrance, ensuring a consistent trajectory. To prevent the MFS-ball from escaping from the right side of the V-shaped cross-sectional track, the CNC program presets a rightward movement limit of 50 mm for the MFS-ball within the track. Upon reaching this travel limit, the MFS-ball is driven by the CNC system to reverse direction and return toward the starting point of motion. As verified through practical testing, when the MFS-ball reaches the extreme right end, it maintains a safety clearance of approximately 15 mm from the right exit of the V-shaped cross-sectional track.

### 2.2. Processing Mechanism

**Figure 4:**
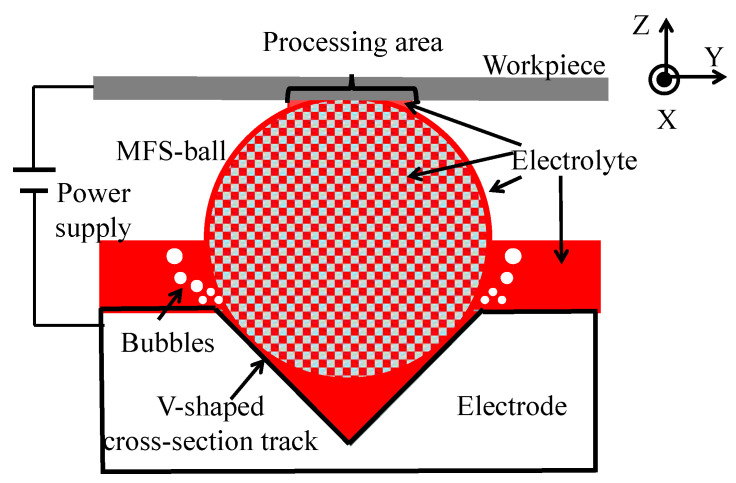
ECM processing diagram view perpendicular to the scanning direction.

Building on previous research results, a diagram of the cross-section of the ECM is presented in Figure 4. During processing, the MFS-ball is placed in the V-shaped cross-sectional track, with the lower part of the ball immersed in the electrolyte and its top in contact with the workpiece surface. The pressure applied by the workpiece to the MFS-ball is approximately 15 N. When the electrical circuit of Figure 1 is turned on, the current flows from the workpiece, through the electrolyte film and the inside electrolyte of the MFS-ball, to the electrode plate. This establishes a closed loop that enables the smooth operation of the ECM process.

On the workpiece surface, the electrolyte is confined to a small area near the contact point between the MFS-ball and the workpiece. During ECM processing, electrochemical reactions occur in the electrolyte distribution area, causing the metal material to corrode. Simultaneously, the generated ECM by-products, which mainly include metal hydroxides like Fe(OH)_2_ and Cr(OH)_3_, adhere to the surface of the MFS-ball. Driven by friction from the reciprocating workpiece, the MFS-ball rolls along the V-shaped cross-sectional track, transporting the ECM by-products to the flowing electrolyte. Then, the by-products are washed away and flow out from the right exit of the V-shaped cross-sectional track. Meanwhile, the fresh electrolyte carried by the MFS-ball is continuously used in subsequent electrochemical reactions.

### 2.3. Experimental Operation for Metal Surface Processing

(1) Workpiece positioning and contact detection testing operation

Begin by connecting all necessary devices, as illustrated in Figure 1, including a power supply, the electrolyte circulation and filtration setup, an oscilloscope, a PC, and a CNC machine. In the ECM section, place the MFS-ball at the starting point on the V-shaped cross-sectional track. Driven by the CNC, move the workpiece downward along the -Z direction until it contacts the top of the MFS-ball, as depicted in Figure 2.

During this movement, connect the MFS-ball and the workpiece to the probes of a digital multimeter (CUSTOM DM-01, CUSTOM Corp., Tokyo, Japan). Upon contact, the electrical circuit of the digital multimeter closes, emitting a beep signal. Subsequently, move the workpiece towards the MFS-ball by an additional 0.1 mm. Based on previous experiments, maintaining the workpiece in contact with the MFS-ball while moving downward by 0.1 mm ensures stability throughout the ECM process.

Choosing a distance of less than 0.1 mm usually results in separation during ECM, as a smaller distance will lead to the pressure between the workpiece and the MFS-ball exceeding 15 N, potentially causing the ball to be crushed by the workpiece. Therefore, a distance of 0.1 mm and a pressure of about 15 N are selected.

(2) Pre-scanning operation

Activate the electrolyte pump to supply clean electrolytes to the ECM section. Simultaneously, the workpiece moves along the preprogrammed trace with the power supply turned off. This operation, referred to as pre-scanning, aims to establish a stable electrolyte film on the MFS-ball surface and accumulate electrolytes in the processing area. The thickness of the electrolyte film, approximately 0.2 mm, is crucial for the stable ECM process, and the influence and necessity of pre-scanning were detailed in our previous study [25].

(3) ECM in metal surface processing

Following the pre-scanning, turn on the power supply. The workpiece and the MFS-ball move repetitively along the V-shaped cross-sectional track. During this stage, the machining current flowing in the circuit induces electrochemical reactions on the workpiece surface, leading to the corrosion of materials through electrolysis.

(4) Ending operation

After ECM, turn off the power supply and electrolyte pumps. Move the workpiece upward in the +Z direction, and switch off the CNC machine.

Common experimental conditions are listed in Table 1, including SUS304 as both the tool electrode and workpiece material, a workpiece moving speed of 10 mm/s, and a 10 wt.% sodium chloride solution as the electrolyte. The reasons for selecting these conditions are as follows:

First, SUS304 is one of the most widely used metal materials in industrial production, making it highly representative. Additionally, its mechanical processing performance presents certain challenges, and choosing this material enhances the industrial reference value of the experimental results.

Second, the workpiece moving speed was set to 10 mm/s, a parameter optimized based on previous experimental findings. The experiments demonstrated that at 10 mm/s, a good balance between processing stability and efficiency could be achieved.

Then, the number of reciprocating pre-scans was determined to be eight times, primarily for efficiency optimization: when the number of passes is less than eight times, the processed groove’s depth is insufficient, leading to less distinct cross-sectional morphology comparisons in subsequent parallel processing. Conversely, exceeding eight times significantly increases processing time, reducing overall efficiency. Therefore, eight reciprocating passes represent the optimal choice that balances groove depth and processing efficiency.

## 3. Establishment of Geometry Model and Simulation of Metal Surface Material Removal

In preceding sections, we introduced the experimental setup and processing operations for subsequent experiments. However, it is impossible to directly observe the ECM process at the atomic and ionic level. Hence, to enhance detailed data and the conditions, in this section, we employed COMSOL Multiphysics for finite element analysis to simulate the ECM processing procedure and results.

### 3.1. Conception and Establishment of ECM Geometry Model

To address the limitation of previous studies that only conducted 2D simulations for partial processing regions, this work developed a 3D model based on the actual CED-ECM process which was shown in Figure 2. The model comprehensively reconstructs the key components of the actual CED-ECM process. First, positioned at the bottom, the tool electrode features a V-shaped cross-sectional track filled with 10% NaCl solution used as the electrolyte of CED-ECM processing; second, the SUS304 workpiece is positioned at the top, connected to the tool electrode via an electrolyte-saturated MFS-ball, with a continuous electrolyte film covering the MFS-ball’s surface. All geometric parameters and material properties in the 3D model are strictly set according to actual CED-ECM processing conditions, ensuring physical consistency between the simulation and the experiment.

Considering that some parameters in actual CED-ECM processing are difficult to measure precisely, this study introduces reasonable simplifications to balance computational efficiency and simulation accuracy. The main simplifications and assumptions are as follows:

① The size and shape of the V-shaped cross-sectional track remain consistent with reality, and both the electrode and workpiece materials are SUS304. In this simulation, the voltage on the workpiece surface is set to 13 V, roughly the same as the voltage value in the actual experiment, while grounding the electrode at 0 V.

**Figure 5:**
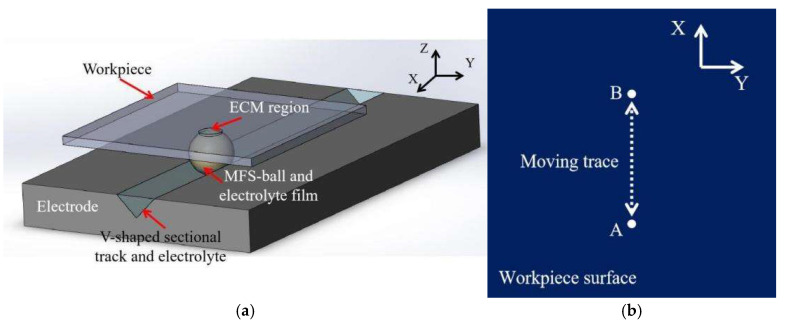
Diagram of 3D model of ECM devices and moving trace on the workpiece surface. (**a**) 3D model of ECM devices. (**b**) Moving trace on the workpiece surface (Point A and Point B are the two end points of moving trace).

② The electrolyte material is a 10 wt.% NaCl solution with conductivity of 12 S/m. Meanwhile, as shown in Figure 5a, for the convenience of modeling and analysis, the electrolyte at the top of the V-shaped cross-sectional track is at the same level as the electrode surface. Upon comparing the results obtained later, this simplification has no impact on the analysis.

③ The MFS-ball absorbs electrolytes through capillary action, but in order to reduce the complexity of the simulation, this process is not included in our simulation. Therefore, the thickness of the electrolyte film on the MFS-ball surface, the area of the workpiece surface covered by the electrolyte, and the amount of electrolyte present in the processing region remain constant throughout the simulation.

④ The material of the MFS-ball is conductive to allow machining currents to pass through it. In the simulation, the conductivity of the MFS-ball is approximately 6 S/m, as obtained from our previous research [26].

⑤ Instead of rolling, the MFS-ball’s movement is simulated as a translation with a speed of 10 mm/s (consistent with actual ECM process parameters) and a distance of 30 mm. This simplification is implemented to reduce the total simulation duration while maintaining kinematic fidelity.

⑥ Several variables, such as electrolyte concentration, flow rate, conductivity, and temperature, are held constant in the simulation, and the by-products of ECM are neglected.

### 3.2. Mutiphysics Formulations

According to electric field theory, the Laplace equation can be used to obtain the potential at any point in the electrolyte domain, and it is given by

(1) $$\nabla^{2}P=0$$

where ∇ represents the Laplace operator and *P* is the potential field. We know that the current density (*i*) of the electrolytic domain can be calculated through the electrical potential (*u*) with Ohm’s law.

(2) $$I=\frac{U}{R}=\frac{U\kappa A}{\Delta }$$

(3) $$i=\frac{I}{A}=\frac{U\sigma }{\Delta }=\sigma \nabla u$$

where *σ* is the electrical conductivity of the electrolyte.

Faraday’s equation was used to govern the material dissolution rate of the ECM process, and is given by

(4) $$V_{n}=\frac{\eta \cdot W\cdot i}{z\cdot F\cdot \rho_{w}}$$

where *η* is the current efficiency, which was set to 100% in this study because the NaCl aqueous solution was used. *V_n_* is the material dissolution rate, *W* is the atomic weight, *z* is the valence of the workpiece material, and *ρ_w_* is the density of the material. These parameters depended on the workpiece material used in the simulations and experiments. *F* is the Faraday’s constant equal to 96,500 C/mol, and *i* is the current density obtained from Equation (3).

During processing, the material dissolution occurs as the MFS-ball moves, and the amount of the material dissolution can be obtained by

(5) $$t=\frac{D}{v}$$

(6) $$M=V_{n}t$$

where *t* is the time of ECM processing, *D* is the total moving distance of the MFS-ball, and *M* is the total amount of material dissolved by the ECM.

The detailed simulation conditions of this study are listed in Table 2.

### 3.3. Simulation Results

**Figure 6:**
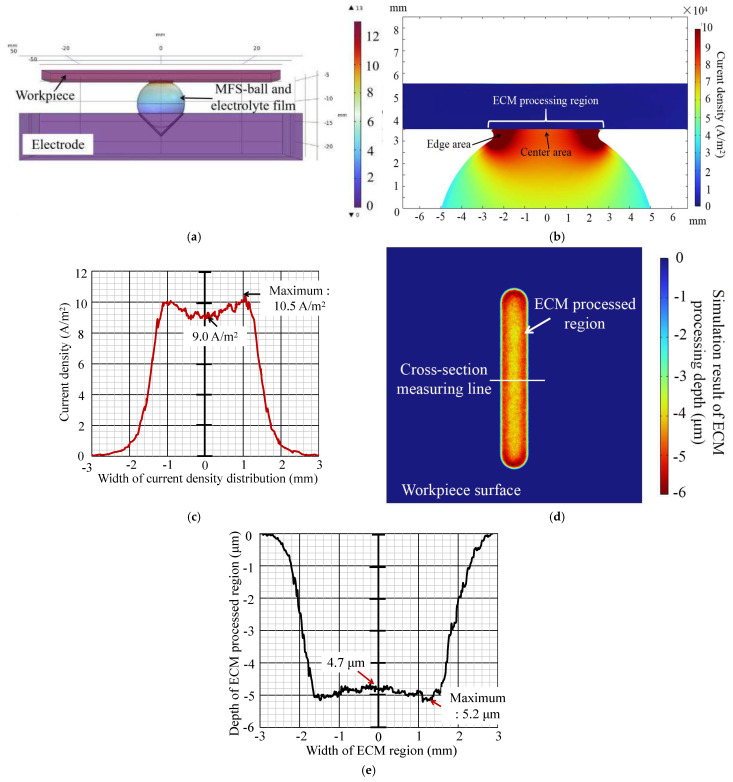
Simulation results of voltage and current density distribution and the ECM processing region. (**a**) Voltage distribution. (**b**) Current density distribution. (**c**) Current distribution curve of the ECM processing region. (**d**) Simulation result of the ECM processing depth. (**e**) Cross-section profile of the ECM processing region.

Using the 3D model of ECM, multiphysics formulations (1)–(6), and simulation parameters in Table 2, this study derived simulation results, presented in Figure 6. For clarity, voltage distribution was observed along the workpiece’s moving direction, and the current density distribution was magnified for a cross-section view of the processing area. To numerically express the current density distribution in the processing area, Figure 6c displays the cross-section curve of the current density distribution at the ECM processing region. Simultaneously, Figure 6d,e present the processed result and its cross-section profile of the white line on the workpiece surface.

As shown in Figure 6b,c, the current density in the processing area rapidly increases from the boundaries to a peak, then gradually decreases toward the center. The edge area closest to the boundary of the processing area exhibits the highest current density, approximately 10.5 A/m^2^, while the center area has a lower current density of approximately 9.0 A/m^2^. Figure 6d,e show similar distribution in the cross-section profile of the ECM processing region, where the depth of the central area (about 4.7 μm) is slightly smaller than that of the edge area (about 5.2 μm).

**Figure 7:**
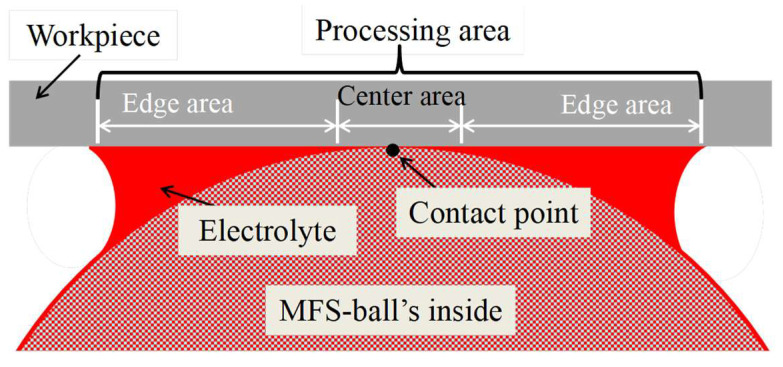
Diagram of the ECM processing area.

The reasons for these results have been summarized from previous studies through observation and actual experimental results, and can be summarized as follows:

① As shown in Figure 7, the center of the ECM processing area, around the contact point between the workpiece and the MFS-ball, has significantly less electrolyte than the edge area. It is found that larger amounts of electrolyte are essential for efficient electrochemical reactions from our previous experiments.

② During ECM, the machining current can flow through the inside electrolyte of the MFS-ball. However, the internal resistance of the MFS-ball is higher than that of the external electrolyte film, due to the reason given in ①. Many more currents flow through the electrolyte film instead of the MFS-ball’s inside. Consequently, the machining current density around the edge area of the ECM processing region is greater than the center area.

③ During ECM processing, a higher machining current density results in a faster electrochemical reaction rate, leading to more material being eroded from the workpiece surface in the same time period, and consequently, a greater ECM processing depth.

In summary, these simulation results align with previous experimental findings and provide a reasonable explanation based on current density distribution. Unlike our previous research, this study not only focused on simulating specific aspects of ECM but also conducted comprehensive simulations throughout the entire processing flow. Therefore, this simulation serves as a crucial guide for future practical experiments, offering valuable references for parameter optimization, process improvement, and other practical aspects during actual processing.

## 4. Metal Surface Processing Method, Results and Verification with Simulation Results

### 4.1. Metal Surface Processing Method and Results

**Figure 8:**
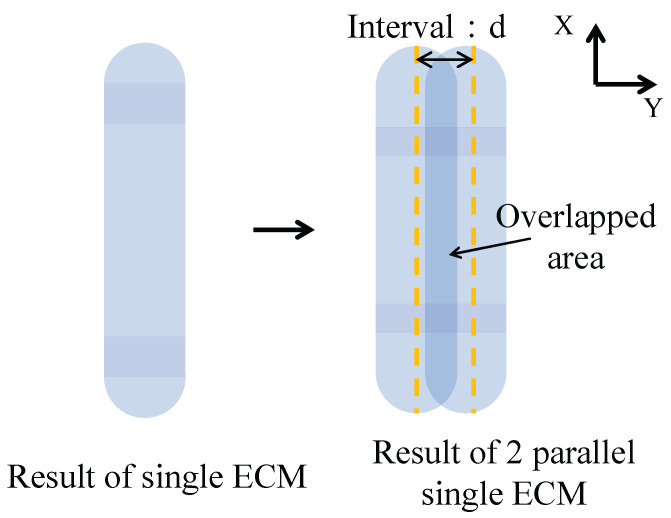
Schematic of a single ECM result and two parallel ECM results.

**Figure 9:**
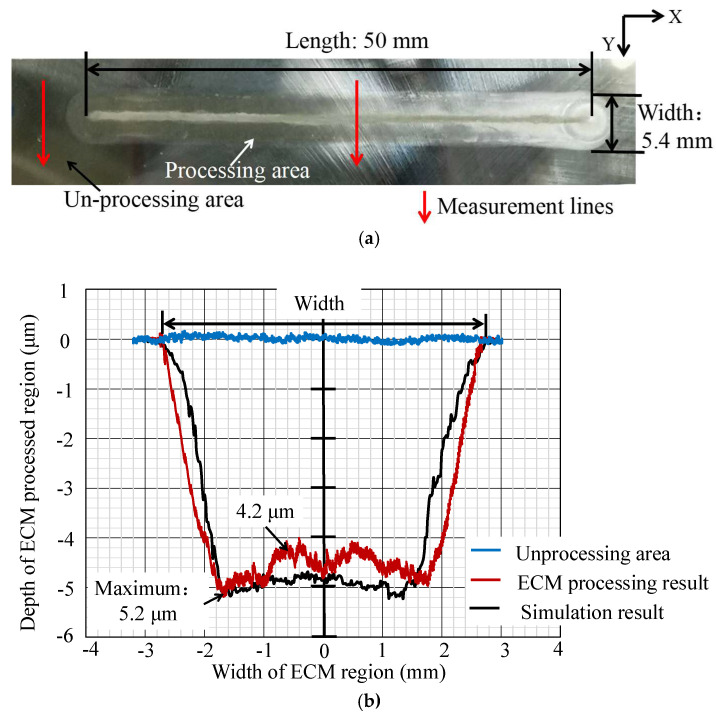
ECM processing result and its measurement result. (**a**) Single ECM processing result on the workpiece surface. (**b**) Measurement result of the ECM processing area and comparison between the simulation results.

To validate the simulation results presented in Section 3, we conducted an actual experiment for metal surface processing following the steps outlined in Section 2.3. In the experiment, as illustrated in Figure 5b, an MFS-ball was driven by the workpiece, performing reciprocal motion between points A and B on the workpiece surface. To increase the length of the processed area, the distance between points A and B needs to be extended. Expanding the width of the processed area requires several additional ECM processes at intervals along the *Y*-axis direction after the initial ECM process. The processing diagram is depicted in Figure 8. Consistent with the simulation results in Figure 6d, this part of the ECM process was only performed once in the actual experiment. Detailed experimental parameters are presented in Table 3, while the processing results and measurement results are shown in Figure 9.

As shown in Figure 9, there is a groove that has been processed using the ECM method on the workpiece surface. The width of the groove is approximately 5.4 mm, the maximum depth at the groove’s edge is approximately 5.2 μm, and the depth at the center of the groove is approximately 4.7 μm.

**Figure 10:**
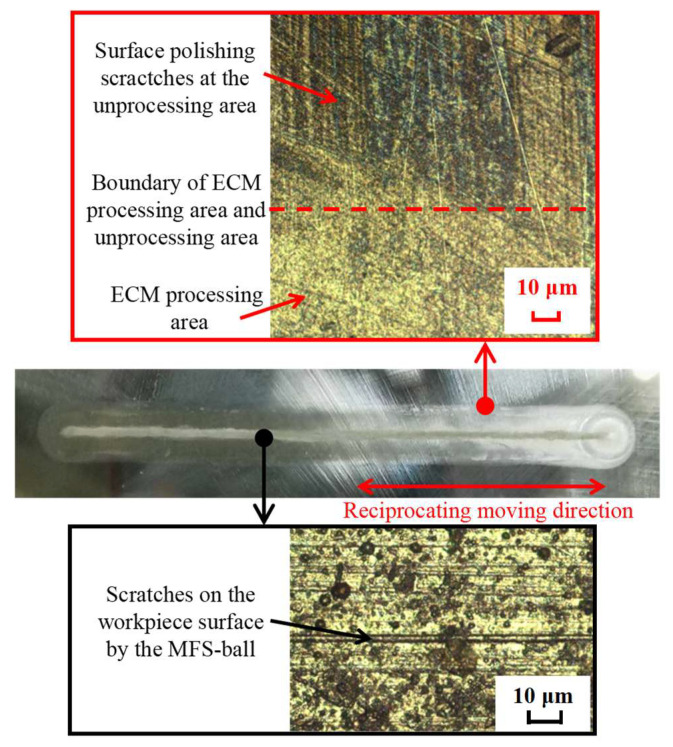
Microscopic observation image of the ECM processing result.

In addition, the ECM processing area was observed under a microscope, and the observation photo is shown in Figure 10. As shown in the red line frame of Figure 10, it is evident that a boundary exists between the unprocessed area, where scratches resulting from surface polishing are present on the workpiece surface, and the ECM processing area, where scratches have been removed through electrochemical reactions during the ECM process. More importantly, there is no stray corrosion at the boundary between the ECM processing area and the unprocessed area, which provides compelling evidence that the CED-ECM method can effectively prevent the occurrence of stray corrosion in ECM.

Furthermore, as shown in the black line frame of Figure 10, in the central area of the ECM processing result, there are a lot of scratches parallel to the reciprocating moving direction of the MFS-ball. These scratches are believed to have formed due to sliding between the MFS-ball and the workpiece surface during the movement process. This finding effectively explains why there is a white trace parallel to the moving direction of the MFS-ball in the center of the processed area, which is the point of contact between the MFS-ball and the workpiece during CED-ECM processing.

### 4.2. Comparison and Discussion of Experiment Result and Simulation Result

In Figure 9a, it is evident that the processed areas in both the actual experiment and simulation exhibit consistent, capsule-like shapes, elongated in nature. Furthermore, the measurement result in Figure 9b indicates that under conditions aligning with the simulation parameters, the cross-section profiles of the actual processed area and simulation results exhibit strong similarities. This strongly confirms the simulation results presented in Section 3. It is clear that, during ECM, a higher current density leads to a higher electrochemical reaction rate, ultimately resulting the corrosion of metal materials in the same duration, and resulting in a deeper processed depth.

However, there are some observed differences, particularly in cross-section profiles of the simulation result and the actual ECM processing result not aligning well. The difference between the ECM processing result and the simulation result and the reasons for the difference are listed as follows:

① The bottom width of the simulation result is approximately 0.6 μm narrower than the ECM processing result, with a more constricted overall profile and a flatter bottom; specifically, the depth variation between the maximum and minimum depths of the simulation result is only about 0.3 μm, while the depth variation at the bottom of the ECM processing result reaches about 1 μm. This indicates that the cross-sectional shape regularity of the ECM processing result is weaker than that of the simulation result.

② These discrepancies primarily arise from neglecting certain conditions existing in the actual ECM process for the purpose of simplifying calculations in the simulation. One crucial aspect is the dynamic changes in the thickness of the electrolyte film and the volume of electrolyte at the ECM processing area. In reality, during ECM processing, the thickness of the electrolyte film and the amount of electrolyte at the processing area are not completely constant due to evaporation caused by temperature rise and the consumption of water in the electrolyte involved in electrochemical reactions. Another critical factor is that the by-product sludge generated during ECM alters the local electrolyte conductivity in the processing region. Consequently, there are variations between the actual processing results and the simulation results.

## 5. Exploration of Metal Surface Processing for Practical Application

### 5.1. Method of Metal Surface Processing by Changing the Interval Value

In our previous study [26], we examined the application of the ECM method for shallow groove scratching and metal marking processing. However, due to the limitation of electrolyte distribution by the MFS-ball, the groove width and English alphabet markings are difficult to adjust. Hence, this section aims to extend metal surface processing to a broader range.

**Figure 11:**
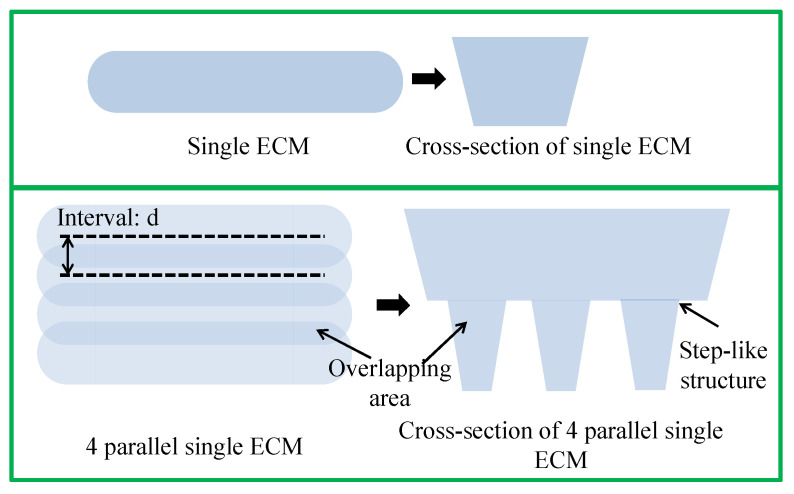
Diagram of overlapping area and cross-section during ECM.

By adjusting the interval between consecutive single ECMs, a wider ECM processing outcome can be achieved. For ease of explanation, Figure 11 illustrates the repetition of a single ECM four times, consistent with the subsequent experiment. When four parallel single ECMs are performed, overlapping occurs in the intersection area covered by multiple single ECMs. The extent of the overlap influences the depth of ECM processing in that region. By manipulating the interval value and the number of parallel single ECMs, it is possible to achieve metal surface processing with various cross-section profiles.

According to Section 3, the width of a single ECM is approximately 5.4 mm. To achieve partial overlapping, the interval value needs to be maintained between 0–5.4 mm. An interval of 0 mm implies that each single ECM is performed at the same location, resulting in complete overlap. When the interval is set to 5.4 mm, single ECMs are positioned parallel to each other, resulting in almost no overlap.

Furthermore, based on our preliminary experiments, an interval value between 0 mm and 0.5 mm yields cross-section profiles with roughly the same shape, except for the width of the processed result. Therefore, the minimum interval value in the subsequent processing is set to 0.5 mm. Several representative experiments with interval values of 0.5 mm, 1.0 mm, 1.5 mm, 2.0 mm, 2.5 mm, and 3.0 mm were selected for visual demonstration. The detailed experimental conditions are outlined in Table 4.

### 5.2. Metal Surface Processing Result for Various Cross-Section Profiles

**Figure 12:**
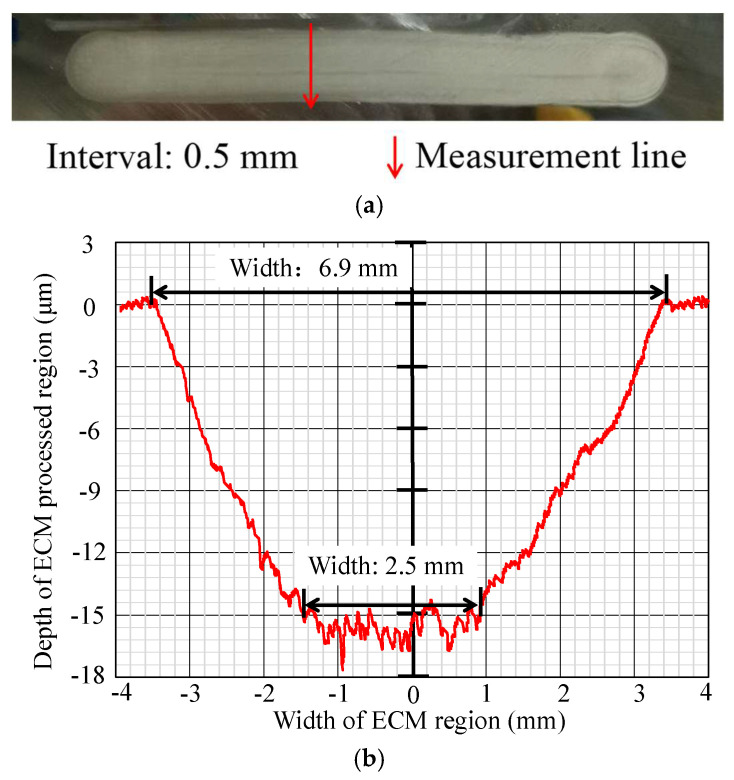
Photo and cross-section profile of the metal surface processing result by ECM with an interval of 0.5 mm. (**a**) Metal surface processing result by ECM. (**b**) Measurement result of cross-section profile.

**Figure 13:**
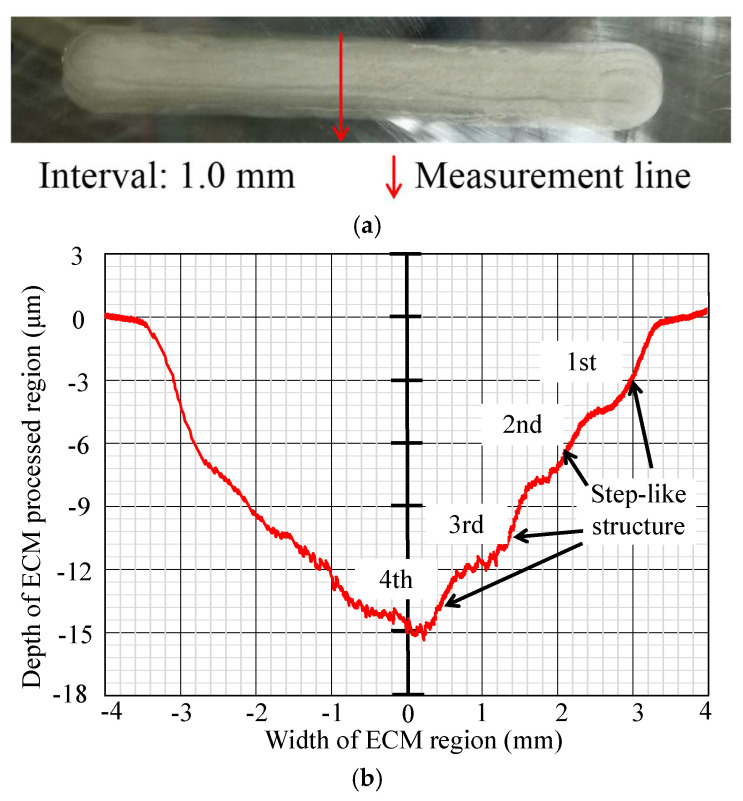
Photo and cross-section of the metal processing result with an interval of 1.0 mm. (**a**) Metal surface processing result by ECM. (**b**) Measurement result of cross-section profile.

**Figure 14:**
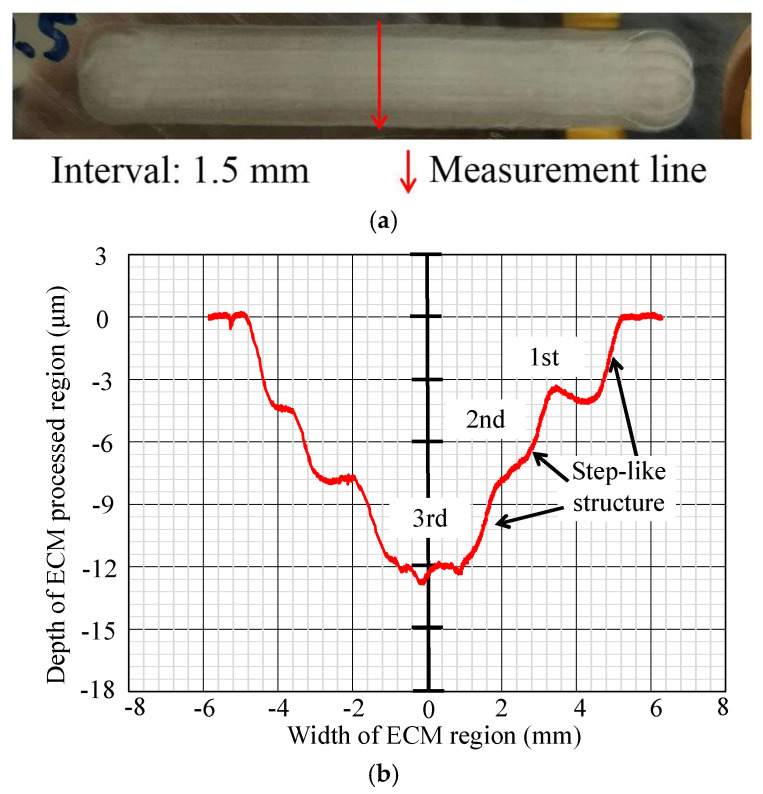
Photo and cross-section of the metal processing result with an interval of 1.5 mm. (**a**) Metal surface processing result by ECM. (**b**) Measurement result of cross-section profile.

**Figure 15:**
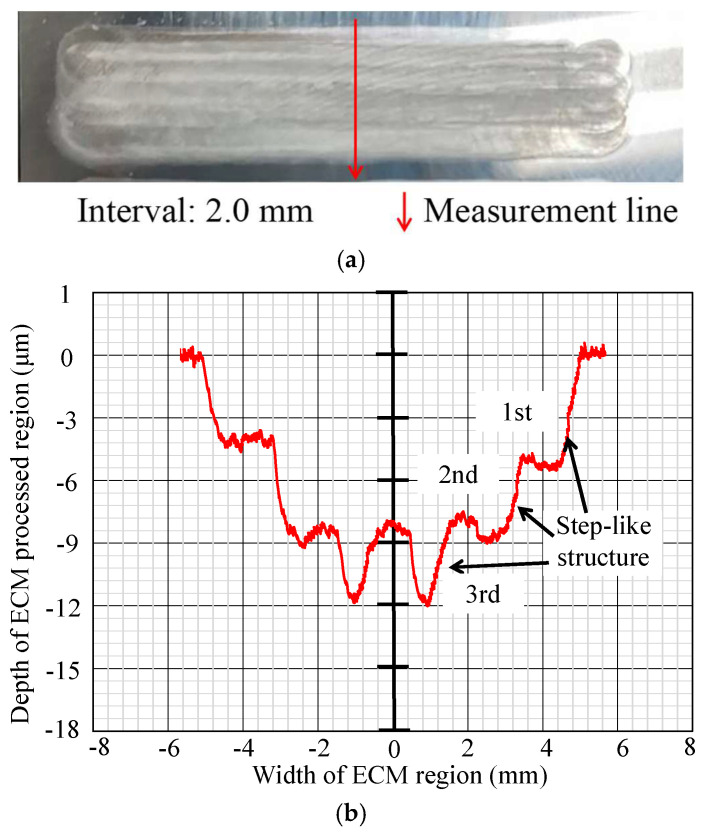
Photo and cross-section of the metal processing result with an interval of 2.0 mm. (**a**) Metal surface processing result by ECM. (**b**) Measurement result of cross-section profile.

**Figure 16:**
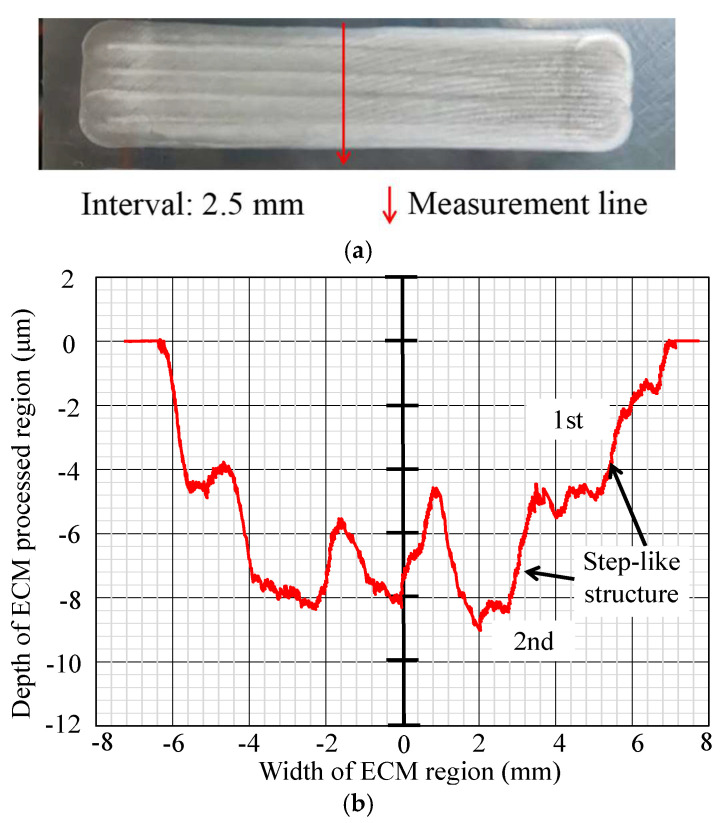
Photo and cross-section of the metal processing result with an interval of 2.5 mm. (**a**) Metal surface processing result by ECM. (**b**) Measurement result of cross-section profile.

**Figure 17:**
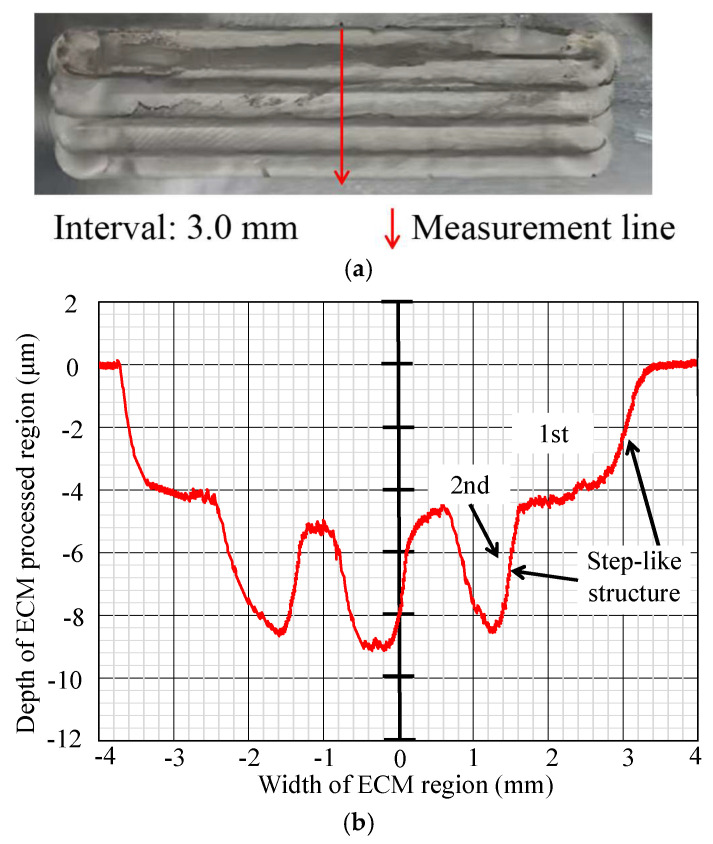
Photo and cross-section of the metal processing result with an interval of 3.0 mm. (**a**) Metal processing result by ECM. (**b**) Measurement result of cross-section profile.

In Figure 12, Figure 13, Figure 14, Figure 15, Figure 16 and Figure 17, photos illustrating the processing results of each metal surface, accompanied by their respective cross-sectional profiles, are presented.

It is evident from Figure 12 that with an interval value of 0.5 mm, the processing result exhibits a U-shaped cross-section profile on the workpiece surface. The processed width of the processed area is approximately 6.9 mm, and the relatively flat bottom, whose depth is about 15–18 μm, has a width of approximately 2.5 mm. As the number of single ECM iterations increases in future practical applications, both the bottom and the width of the processed area will increase to obtain a wider metal surface processing.

Subsequently, Figure 13, Figure 14 and Figure 15 reveal that as the interval values increase to 1 mm, 1.5 mm, and 2 mm, the processed area’s width expands, while the maximum depth decreases. This is due to the consistent number of processing times and duration, causing the maximum depth to diminish as the processing area expands. Simultaneously, a step-like structure appears at the sidewalls of the cross-section profile, with the number of step-like structures being four, three, and three, respectively. The first step-like structure, which is close to the workpiece surface, indicates exclusive corrosion during the initial single ECM, remaining unaffected by subsequent procedures. The second step-like structure, which is below the first step-like structure, indicates overlapping between the initial and secondary single ECMs, while remaining uncovered during the third single ECM and the fourth single ECM. The following step-like structures have a similar formation mechanism.

Figure 16 and Figure 17 demonstrate that when the interval is 2.5 mm or 3 mm, the overlapping area diminishes further, with a consistent number of step-like structures at two.

**Figure 18:**
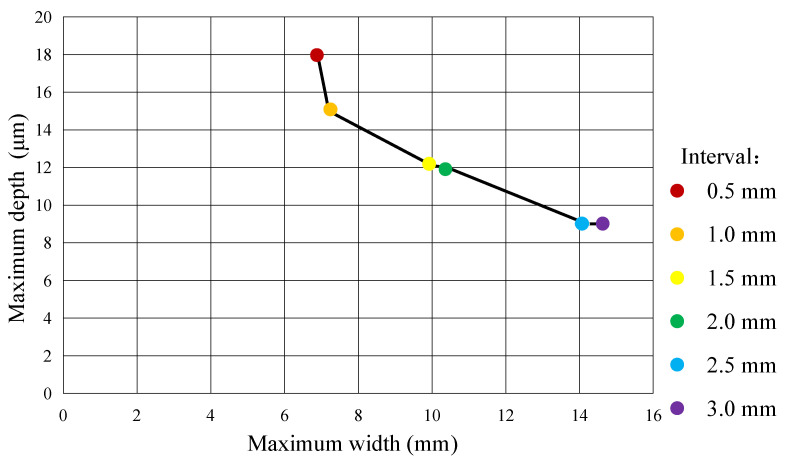
Distribution plots of maximum depth and maximum width of metal processing results with different intervals.

To clearly demonstrate the differences in processing results under varying interval values, we generated comparative distribution plots, as shown in Figure 18, based on the maximum depth and maximum width of processed grooves from Figure 12, Figure 13, Figure 14, Figure 15, Figure 16 and Figure 17. As clearly observed from Figure 18, with the increase of the interval value, the maximum width of the CED-ECM processing result gradually increases, while the maximum depth correspondingly decreases. It is thought that, as the interval value increases, the width of the overlapping area of the CED-ECM processing traces narrows, and the CED-ECM processing time of the overlapping area decreases, leading to a reduction in the maximum depth value of the CED-ECM processing result.

In conclusion, based on the experimental results, adjusting the interval value allows the ECM method to achieve diverse results with different cross-sectional profiles, processing widths and depths, thus meeting various metal surface processing requirements.

## 6. Conclusions

In this study, we have developed a method for metal surface processing, expanding upon our previous ECM research. Through rigorous research involving simulation prediction, experimental verification, and practical application, we conducted a comprehensive examination of the ECM method. The main conclusions are as follows:

(1) The simulation results using COMSOL Multiphysics provided valuable data that are challenging to directly observe during the ECM process, such as voltage and current density distributions in the ECM region. The simulation successfully predicted the ECM processing result on the workpiece surface.

(2) By comparing the experimental results with the simulation results, we confirmed the authenticity and reliability of the simulation results. The simulation results revealed the reason for the difference in depth between the center and edge of the ECM processing area.

(3) Adjusting the processing interval value allowed for the achievement of different cross-section profiles on the metal surface, providing an ECM method for future metal surface processing.

Above all, the CED-ECM method demonstrates significant advantages in metal surface processing. By controlling the processing area, it can achieve processed grooves with diversified cross-sectional shapes, while the process is strictly confined to the target area, effectively avoiding stray corrosion in unneeded processing areas. This characteristic grants it unique application value in precision manufacturing. On one hand, it can specifically remove the recast layer and micro-cracks at specific locations on the workpiece surface after EDM processing, addressing local defects that are difficult to handle with traditional methods. On the other hand, it shows outstanding adaptability in processing oil pockets on the surface of metal friction pairs. By regulating CED-ECM processing parameters, this method enables precise control over the depth, size, and cross-sectional morphology of the oil pockets, thus meeting the differentiated requirements for the depth, size, and shape of oil pockets across different friction pair positions. This provides a reliable process solution for enhancing the lubrication performance of friction pairs.

## Figures and Tables

**Table 1:** Common experimental conditions.

Item	Specification
Absorption material	MFS-ball
Workpiece	SUS304
Electrode	SUS304
Moving speed of workpiece	10 mm/s
Electrolyte	10 wt.% NaCl solution
Temperature of electrolyte	20 °C
Number of reciprocating pre-scans	8 times

**Table 2:** Simulation conditions.

Item	Specification
Diameter of MFS-ball	9.40 mm
Thickness of electrolyte film	0.2 mm
Width of ECM processing area	5.4 mm
Potential of workpiece	13 V
Potential of electrode	0 V
Electrical conductivity of electrolyte film	12 S/m
Electrical conductivity of MFS-ball	6 S/m
Temperature	293.15 K
Reciprocating times of ECM during simulation	8 times

**Table 3:** Experimental parameters of the single ECM process.

Item	Specification
Diameter of MFS-ball	9.40 mm
Tolerance of MFS-ball diameter	+0.05 mm/–0 mm
Distance of movement of single ECM	50 mm
Constant current	40 mA
Reciprocating times during single ECM	8 times

**Table 4:** Experimental parameters of 4 parallel single ECM.

Item	Specification
MFS-ball diameter	9.4 mm
Tolerance of MFS-ball diameter	+0.05 mm/–0 mm
Distance of movement of single ECM	50 mm
Constant current	40 mA
Reciprocating times of each single ECM	8 times
Interval value	0.5, 1.0, 1.5, 2.0, 2.5, 3.0 mm

## Data Availability

The datasets generated and supporting the findings of this article are obtainable from the corresponding author upon reasonable request.
